# Supplementary material for: WordCommentsAnalyzer: A windows software tool for qualitative research
Source: F1000Res. 2018 Sep 4;7:536. Originally published 2018 May 3. [Version 2] doi: 10.12688/f1000research.14819.2 (PMC6137411; doi:10.12688/f1000research.14819.2)
Supplement: Supplementary file 3 [file f1000research-7-17614-s0002.tgz › b2a79496-4ada-47c4-9682-a99ce867836d.zip!/Eight Iranian Health Journals/Journal of Research in Rehabilitation Sciences _48.docx]

© Journal of Research in Rehabilitation Sciences . This work is licensed under CC BY-NC 4.0 (https://creativecommons.org/licenses/by-nc/4.0/).

Volume & Issue: دوره 5، شماره 2: 1388

Title: تأثیر تمرینات هوازی بر پارامترهای بیوشیمیایی زنان مبتلا به سندرم تخمدان پلی‌کیستیک

http://jrrs.mui.ac.ir/index.php/jrrs/article/view/114

چکیده مقدمه: هدف تحقیق حاضر بررسی تأثیر تمرینات هوازی بر پارامترهای بیوشیمیایی زنان مبتلا به سندرم تخمدان پلی‌کیستیک بود. مواد و روش‌ها: جهت اجرای این تحقیق نیمه تجربی، 24 زن مبتلا به سندرم تخمدان پلی‌کیستیک به طور تصادفی انتخاب و به دو گروه 12 نفری تقسیم شدند. برنامه تمرینی هوازی در طول دوازده هفته بر روی گروه تجربی اجرا شد. داده‌های کلینیکی همچون مورفولوژی تخمدان‌ها، درصد BF (Body Fat)، WC (Waist Circumferance)، WHR ((Waist to Hip Ratio و سطح هورمون‌های سرم مانند انسولین، آندروستندیون، تستوسترون DHEA-S و SHBG قبل و بعد از اجرای پروتکل تمرینی اندازه گیری شد. برای تجزیه و تحلیل آماری از آزمون‌های t زوج و مستقل استفاده شد (05/0 ≥ α). یافته‌ها: آزمودنی‌های تحقیق دارای میانگین سن 77/3 ± 7/22 سال، قد 29/5 ± 87/159 سانتی‌متر، وزن 12/14 ± 88/69 کیلوگرم و شاخص توده بدنی 02/5 ± 53/27 کیلوگرم بر متر مربع بودند. سه ماه پس از اجرای برنامه تمرینی هوازی، کاهش معنی‌داری در اندازه تخمدان (تخمدان راست با 001/0 = P و تخمدان چپ با 002/0 = P)، درصد BF (004/0 = P)، WC (002/0 = P)، WHR (01/0 = P)، انسولین ناشتا (0004/0 = P) و آندروستندیون (01/0 = P) مشاهده شد. تغییر معنی‌داری در تستوسترون سرم (53/0 = P)، سطوح DHEA-S (16/0 = P) و SHBG یا Hormone Binding Globulin Sex  (40/0 = P) یافت نشد. بحث: از ساز و کارهای مربوط به کاهش حجم تخمدان می‌توان به ایجاد محیط آندوکرینی مطلوب به دنبال افزایش SHBG، کاهش آندروژن‌های آزاد و بهبود حساسیت به انسولین اشاره کرد. احتمال می‌رود در بیماران مبتلا به سندرم پلی‌کیستیک، کاهش در حجم تخمدان موجب کاهش آندروستندیون در گردش شده، علائم کلینیکی و پارامترهای بیوشیمیایی را بهبود بخشد.

Title: تأثیر اضافه نمودن يك وظيفه ثانويه شناختي بر متغيرهاي كينماتيكي راه رفتن در بيماران مبتلا به پاركينسون

http://jrrs.mui.ac.ir/index.php/jrrs/article/view/107

چکیده مقدمه: بیماری پارکینسون با ضعف کنترل وضعیت بدن (Posture) و افت عملکرد حرکتی، به ویژه در راه رفتن، همراه است. شناسایی عواملی که در بهبود عملکرد حرکتی این بیماران مؤثرند از اهمیت کلینیکی برخوردار است. هدف از انجام این تحقیق، ارزیابی تأثیر یک وظیفه شناختی ثانوی بر متغیرهای کینماتیک راه رفتن در بیماران مبتلا به پاركينسون بود. مواد و روش‌ها: تعداد 10 بیمار زن مبتلا به پارکینسون تحت درمان با لوودوپا به ترتیب با میانگین سن، قد و وزن 6/3 ± 6/59 سال، 4/4 ± 2/157 سانتی‌متر و 7/6 ± 2/67 کیلوگرم به عنوان گروه تجربی و 14 زن سالم غیر ورزشکار با سن، قد و وزن مشابه به عنوان گروه شاهد در این تحقیق شرکت کردند. الگوی کینماتیک راه رفتن آزمودنی‌ها در حالت عادی و همراه با یک وظیفه شناختی ثانوی با استفاده از دستگاه تجزیه و تحلیل حرکتی (High-Rez Motion Analysis System) مورد اندازه گیری قرار گرفت. طرح آزمایش شامل یک عامل بین گروهی و دو عامل درون گروهی (هر یک با دو سطح) بود و برای تحلیل آماری از آزمون Repeated Measure (05/0 = µ) استفاده گردید. یافته‌ها: طول گام (Stride) افراد سالم و بیمار در راه رفتن عادی به ترتیب 7/4 ± 9/121 و 1/6 ± 7/79 و در راه رفتن همراه با اجرای وظیفه ثانوی شناختی 0/5 ± 3/127 و 5/6 ± 4/86 بود. در بیماران مبتلا به پارکینسون، طول گام، سرعت گام برداری، سرعت زاویه‌ای مفاصل اندام تحتانی، و دامنه حرکتی مفاصل اندام تحتانی کوچک‌تر از افراد سالم بود. اما، زمان حمایت دوگانه در بیماران به طور معنی‌داری بیشتر از این مقدار در افراد سالم بود (001/0 = P). میزان طول گام بیماران و دامنه حرکتی مفصل ران در راه رفتن با وظیفه ثانوی شناختی به طور معنی‌داری بهبود یافت (001/0 = P). بحث: وظیفه ثانوی شناختی هنگام راه رفتن موجب بهبود متغیرهای کینماتیکی راه رفتن می‌شود. افزودن وظیفه ثانوی شناختی هنگام تمرین راه رفتن در برنامه توان‌بخشی بیماران مبتلا به پارکینسون توصیه می‌گردد.

Title: تأثیر چسب زني استخوان پاتلا (Patellar taping) بر فعاليت الكتريكي ماهيچه‌هاي Vasti در افراد مبتلا به سندرم درد مفصل پاتلوفمورال (PFPS) حين حركت خمیدگی یک‌طرفه زانو

http://jrrs.mui.ac.ir/index.php/jrrs/article/view/108

چکیده مقدمه: یکی از شایع‌ترین اختلالات اسکلتی-عضلانی ناحیه زانو در بالغین، سندرم درد مفصل پاتلوفمورال (PFPS) مي‌باشد. چسب زنی ناحیه پاتلا ((Patellar taping روش درمانی مؤثری جهت تسکین علایم دردناك در طیف وسیعی از این بیماران است؛ گرچه مکانیسم‌ کاهش علایم پس از استفاده از چسب به درستی مشخص نشده است. هدف از اجراي اين پژوهش ارزيابي فعاليت EMG در ماهيچه‌هاي Vasti پس از استفاده از چسب پاتلا و يافتن مكانيسم احتمالي كاهش درد پس از آن بود. مواد و روش‌ها: در يك پژوهش شاهد- موردی، دو پارامتر اختلاف زماني آغاز فعاليت ماهيچه‌هاي VMO و VL (VMO-VL onset) و مقدار فعالیت نسبي این ماهيچه در 18 بيمار مبتلا به سندرم درد مفصل پاتلوفمورال و 18 فرد سالم، توسط يك دستگاه ثبت امواج الكتريكي ماهيچه (EMG) اندازه گيري گرديد. پارامترهاي مورد اشاره از زانوی آسیب دیده افراد مبتلا قبل از اعمال چسب، در حین اتصال چسب به ناحیه زانو و پس از طی یک دوره چسب زنی (2 تا 5 هفته) و نیز از زانوی سمت سالم بیماران و همچنین هر دو زانوی افراد گروه شاهد در خلال مرحله انقباض اكسنتريك (Eccentric) حرکت خمیدگی یک طرفه زانو (Single -leg squatting) به ثبت رسيد. یافته‌ها: اختلاف معني‌داري بين ميانگين عددي VMO-VL onset در بين افراد گروه شاهد و مبتلايان به PFPS پيش از اقدام به چسب زني به چشم خورد (05/0 > P). همچنین میانگین عددیVMO-VL onset  پيش از اعمال چسب بر روي استخوان پاتلا (54/2 ميلي‌ثانيه، 35/4 = SD)، کاهش معنی‌داری پس از يك دوره چسب زني (00/6- ميلي‌ثانيه،40/3 = SD) پیدا کرد (05/0 > P). با اين وجود هيچ گونه اختلاف معنی‌داري بين مقدار فعالیت نسبی ماهیچه‌های VMO و VL مربوط به زانوی آسیب دیده بیماران مبتلا بهPFPS ، پاي سالم آنان و همچنین افراد سالم گروه شاهد بین وضعيت‌هاي گوناگون چسب زني مشاهده نگرديد (05/0 < P). بحث: کاسته شدن از مقادير عددي VMO-VL onset، كه به معناي انقباض سریع‌تر ماهيچه VMO نسبت به VL تلقي مي‌گردد، ممكن است در اصلاح وضعيت و راستاي استخوان پاتلا (Realignment) به عنوان يك عامل كمكي مطرح باشد. بنابراين شايد بتوان مكانيسم كاهش درد پس از استفاده از چسب پاتلا را با ايجاد تغيير در الگوي فعاليت زماني دو ماهيچه VMO و VL در بيماران مبتلا به سندرم درد مفصل پاتلوفمورال توضيح داد.

Title: بررسی مقایسه‌ای تأثیر روش منیپولاسیون مایوفاشیال با فیزیوتراپی مرسوم در درمان سردرد سرویکوژنیك

http://jrrs.mui.ac.ir/index.php/jrrs/article/view/109

چکیده مقدمه: سردرد سرویکوژنیک (با شیوع 4/0 تا 25 درصد) یک نوع سردرد مزمن یک طرفه است که از ستون فقرات گردنی یا بافت‌های نرم اطراف گردن منشأ گرفته، به سمت اوکولو-فرونتو-تمپورال گسترش می‌یابد. وجود دیدگاه‌های مختلف درمانی بیان‌گر آن است که تنها یک درمان مؤثر برای این سردرد وجود ندارد. در این مطالعه دو روش این بیماری مورد بررسی قرار گرفت. مواد و روش‌ها: مطالعه حاضر از نوع آینده‌نگر، شبه‌تجربی، یک سویه‌کور و تصادفی بود. از بین بیماران حائز شرایط ورود به مطالعه، 44 بیمار به صورت تصادفی انتخاب و به صورت تصادفی وارد دو گروه درمانی فیزیوتراپی مرسوم و منیپولاسیون مایوفاشیال شدند. اطلاعات مربوط به شدت، فرکانس و مدت زمان سردرد توسط پرسش‌نامه و ناتوانی این بیماران توسط شاخص ناتوانی در اثر گردن درد (NDI) قبل و بعد از درمان و پس از پی‌گیری یک ماهه جمع‌آوری شد. سپس اطلاعات توسط آزمون‌های t و t زوج شده مورد تجزیه و تحلیل قرار گرفت. یافته‌ها: در روش منیپولاسیون مایوفاشیال تفاوت معنی‌داری بین شدت (001/0 > P)، فرکانس (001/0 > P) و مدت زمان سردرد (001/0 > P) و ناتوانی بیماران (001/0 > P) در زمان قبل و بعد از درمان وجود داشت. در روش فیزیوتراپی مرسوم تفاوت معنی‌داری بین شدت (001/0 > P)، فرکانس (001/0 > P) و مدت زمان سردرد (005/0 > P) و ناتوانی بیماران (001/0 > P) در زمان قبل و بعد از درمان وجود داشت. بین میانگین فرکانس و مدت زمان سردرد و ناتوانی بیماران در زمان پی‌گیری در دو گروه منیپولاسیون مایوفاشیال و فیزیوتراپی مرسوم تفاوت معنی‌داری وجود نداشت (001/0 < P). بین میانگین شدت درد در زمان پی‌گیری در دو گروه منیپولاسیون مایوفاشیال و فیزیوتراپی مرسوم تفاوت معنی‌داری وجود داشت (05/0 > P). بحث: نتایج حاصل از این مطالعه نشان داد که هر دو روش منیپولاسیون مایوفاشیال و فیزیوتراپی مرسوم در درمان و تسکین علایم سردرد سرویکوژنیک مؤثر می‌باشند ولی در پی‌گیری یک ماهه، منیپولاسیون مایوفاشیال دارای اثرات بهتر و پایدارتر نسبت به فیزیوتراپی مرسوم می‌باشد.

Title: مقايسه تأثیر شش هفته تمرين مقاومتي- تعادلي، سرعتي- تعادلي و تعادلي بر تعادل پوياي مردان سالمند فعال

http://jrrs.mui.ac.ir/index.php/jrrs/article/view/110

چکیده مقدمه: پیش‌گیری و غلبه بر ناتوانایی‌های سالمندان، شناخت مشکلات و نیز بررسی عوامل مؤثر در بهبود کیفیت زندگی آن‌ها موضوع مهمی است. هدف از انجام تحقيق حاضر، مقايسه تأثیر شش هفته تمرين مقاومتی- تعادلی، سرعتی- تعادلی و تعادلی بر تعادل پوياي مردان سالمند فعال بود. مواد و روش‌ها: تعداد 34 نفر از افراد سالمند بدون سابقه آسیب در اندام تحتانی که هفته‌ای سه بار در فعالیت‌های آمادگی جسمانی شرکت داشتند، به صورت داوطلبانه در این مطالعه شرکت كردند. آزمودنی‌های تحقیق به صورت تصادفی به چهار گروه تقسیم شدند. گروه اول تمرینات ترکیبی مقاومتی- تعادلی، گروه دوم تمرینات ترکیبی سرعتی- تعادلی و گروه سوم تنها تمرینات تعادلي را به مدت شش هفته و هر هفته دو جلسه انجام دادند. گروه چهارم نیز به عنوان گروه شاهد در هیچ گونه برنامه تمرینی شرکت نکرده، به برنامه‌های معمول خود ادامه دادند. برای برآورد تعادل پویای آزمودنی‌ها، پس از گرم کردن (10-5 دقیقه کشش)، از آزمون تعادل ستاره (Star Excursion Balance Test یا SEBT) استفاده شد. روز بعد از اتمام دوره تمريني، تعادل پوياي آزمودني‌ها دوباره اندازه‌گيري شد. براي تجزيه و تحليل آماري از روش‌هاي آمار توصيفي، تحليل واريانس يک‌سويه و آزمون تعقيبي توکي در سطح معني‌داري 05/0 ≥ α استفاده شد. یافته‌ها: فاصله دستيابي در هشت جهت SEBT در گروه‌های تمرين مقاومتی- تعادلی، سرعتی- تعادلی و تعادلی، پس از اعمال تمرینات، افزايش معني‌داری یافت. همچنين مشاهده شد که تمرين سرعتی- تعادلی نسبت به تمرين مقاومتی- تعادلی و تعادلی باعث بهبود بيشتري در تعادل پويا مي‌شود. بحث: با توجه به نتايج، استفاده از تمرينات مقاومتی- تعادلی، سرعتی- تعادلی و تعادلی (به ویژه سرعتی- تعادلی) به منظور کاهش احتمال سقوط و بهبود تعادل پوياي افراد سالمند توصیه می‌شود.

Title: بررسي پايايي آزمون‌هاي عملكردي زانو در افراد مبتلا به سندرم درد كشككي- راني

http://jrrs.mui.ac.ir/index.php/jrrs/article/view/111

چکیده مقدمه: سندرم درد كشككي- راني واژه كلينيكي شايعي جهت توصيف شرايط پاتولوژيك درد موجود بين کشکک و كنديل‌هاي ران است. هدف پژوهش حاضر، بررسي پايايي آزمون‌های عملکردي زانو در افراد مبتلا به سندرم درد كشككي- راني بود. مواد و روش‌ها: جهت اجرای این پژوهش، 15 آزمودنی مبتلا به درد کشكکي- راني و 17 آزمودنی بدون درد کشککی- رانی انتخاب شدند و آزمون‌های عملکردی پرس تک‌پايي، پايين آمدن از پله، Squat (چمباتمه زدن) يك‌طرفه و جهش محوري بر روی آن‌ها اجرا شد. برای تجزیه و تحلیل داده‌ها از روش‌هاي آماري آزمون همبستگي Pearson، t زوج و مستقل و ضريب همبستگي درون گروهي ((ICC استفاده گردید (05/0 ≥ α). یافته‌ها: پايايي آزمون‌هاي عملکردي مورد استفاده در این تحقیق در دامنه 79/0 تا 94/0 قرار داشت. در افراد داراي درد کشكکي- راني در تکرار تعداد آزمون‌ها، به جز چمباتمه، اختلاف معنی‌داري یافت شد (01/0 = P). همبستگي معنی‌داري بين چهار آزمون عملکردي، به جز چمباتمه، با درد وجود داشت. شاخص تقارن عضوي (LSI) در گروه اول 80 و در گروه دوم 97 درصد گزارش گردید. در آزمون‌هاي عملکردي پايايي بالايي به دست آمد که با تغييرات درد همبستگي داشت. بحث: با توجه به این که آزمون‌های بررسی شده در این تحقیق دارای پایایی بالایی بودند، از این رو توصیه می‌شود که از این آزمون‌ها برای ارزیابی افراد مبتلا سندرم درد کشککی- رانی استفاده گردد.

Title: اثر ویبریشن بر پیش‌گیری از کوفتگی عضلانی تأخیری در دختران فعال

http://jrrs.mui.ac.ir/index.php/jrrs/article/view/112

چکیده مقدمه: کوفتگی عضلانی تأخیری که بعد از ورزش‌های اکسنتریک رخ می‌دهد، موجب کاهش کارایی و عملکرد ورزشی به خصوص بین ورزشکاران حرفه‌ای می‌گردد. به همین دلیل کاهش این عوارض همواره مورد توجه مطالعات مختلف قرار داشته است. هدف از تحقیق حاضر تعیین میزان تأثیر ویبریشن بر پیش‌گیری از کوفتگی عضلانی تأخیری (Delayed Onset Muscle Soreness یا DOMS) ایجاد شده در نتیجه تمرین اکسنتریک، با فرض کاهش میزان کوفتگی عضلانی تأخیری در اثر تمرین ویبریشن، بود. مواد و روش‌ها: جامعه آماری این تحقیق را 40 نفر زن ورزشکار در دامنه سنی 25-19 سال تشکیل می‌دادند که دارای3 سال سابقه ورزشی بودند. داوطلبان به طور تصادفی در یکی از دو گروه بیست نفره Vibration Training (VT) و Non Vibration Training (Non-VT) قرار گرفتند. حداکثر قدرت ارادی عضلات چهارسررانی (Quadriceps) پای راست و چپ، آستانه درد فشاری در 5، 10 و 15 سانتی‌متر بالای پاتلا و همین طور در وسط عضلات شکمی ساق (Gastrocnemius) هر دو پا و دامنه حرکتی مفصل زانو (زاویه خم شدن و باز شدن زانو) در هر دو پا اندازه‌گیری و ثبت شد. در گروه VT از دستگاه ویبراتور با فرکانس HZ 50 برای اعمال لرزش عضلات چهارسر رانی، شکمی ساق و همسترینگ‌های (Hamstrings) هر دو پا هر یک به مدت 1 دقیقه استفاده شد. سپس هر دو گروه به تمرین اکسنتریک با دستگاه Leg Press پرداختند. 24 ساعت پس از انجام ورزش اکسنتریک، علاوه بر تکرار اندازه‌گیری‌های فوق، سطح درد و آزردگی عضلانی با استفاده از مقیاس Visual Analogue Scale (VAS) مورد ارزیابی قرار گرفت. یافته‌ها: مقایسه میانگین تغییرات پارامتر‌های اندازه‌گیری شده بیانگر کاهش معنی‌دار حداکثر انقباض ارادی عضله چهارسر رانی (05/0 > P) و کاهش معنی‌دار آستانه درد فشاری (05/0 > P)، همچنین کاهش معنی‌دار دامنه حرکتی مفصل زانو (05/0 > P) در گروه Non-VT در مقایسه با گروه VT بود. میانگین سطح ادراک درد عضلانی در 24 ساعت پس از فعالیت اکسنتریک به طور معنی‌داری در گروه VT پایین‌تر از گروه Non-VT بود (05/0 > P). بحث: مقایسه نتایج به دست آمده بین دو گروه VT و Non-VT نشان داد که استفاده از VT می‌تواند به طور قابل ملاحظه‌ای موجب کاهش کوفتگی عضلانی تأخیری ناشی از انجام ورزش‌های اکسنتریک در ورزشکاران گردد.

Title: مروری بر روش‌‌های بازآموزي حسي، به دنبال ترمیم اعصاب محیطی اندام فوقانی

http://jrrs.mui.ac.ir/index.php/jrrs/article/view/113

چکیده مقدمه: به‌ دنبال جراحي ترمیمی تنه اعصاب محيطي در اندام فوقانی، به‌ سرعت يك سازمان‌دهي مجدد کارکردي در مغز اتفاق مي‌افتد؛ اين پديده به ‌صورت شكل‌گيري تغييراتي در نقشه كورتيكال دست مي‌باشد و علت آن انحراف آكسون‌هاي در حال رشد از مسير اوليه و اصلي خود به ‌سمت اندام‌هاي هدف است. برنامه‌هاي بازآموزي حسي، در فاز توان‌بخشي به ‌منظور تسهيل و تأثير گذاري مثبت بر روي روند يادگيري مجدد حسي انجام مي‌شود. اين برنامه‌ها موجب تطبيق دادن سازمان‌دهي سيناپتيك جديد و ارتقای سطح بهبودي حساسيت‌پذيري کارکردي مي‌گردد كه «اگنوزي لمسی» (Tactile Agnosia) نام دارد. هرچند در اغلب موارد، به ‌ویژه در بزرگسالان، نتایج مأيوس‌كننده بوده، روش‌هاي برنامه بازآموزي حسي در چندين دهه اخير تغييري نداشته است. مواد و روش‌ها: در این مطالعه ابتدا به جمع آوری مقالات مرتبط موجود از طریق جستجو در پایگاه‌های اطلاعاتی چون google، Ovid و Science Direct و همچنین مکاتبه با نویسندگان مقالات جهت احراز مقالات پرداخته شد و سپس به طور دقیق، بررسی و مرور مفاهیم و روش‌های موجود انجام پذیرفت. بحث: در اين بررسي، به مرور مفاهيم موجود در علوم اعصاب و شناختي، معرفي يك دليل منطقي براي نياز به تغيير و تعديل روش‌هاي بازآموزي حسي قديمي و پيشنهاد روش‌هاي جديد براي ارتقای ميزان يادگيري مجدد حسي پس ‌از ترميم عصب پرداخته ‌شد.
